# DEVELOPING A DEMENTIA-FRIENDLY COMMUNITY GLOBAL TOOLKIT: INSIGHTS FROM STAKEHOLDERS IN WHO MEMBER COUNTRIES

**DOI:** 10.1093/geroni/igz038.1669

**Published:** 2019-11-08

**Authors:** Fei Sun, Emmanuel Chima, Katrin Seeher, Tarun Dua, Devora Kestal

**Affiliations:** 1 Michigan State University, East Lansing, Michigan, United States; 2 World Health Organization, Genève, Geneve, Switzerland

## Abstract

Drawing on perspectives from stakeholders involved in dementia friendly initiatives (DFIs) in WHO member countries, this paper describes the characteristics of DFIs around the world and summarizes success factors and barriers to implementation. Data were collected through an online consultation survey of 129 stakeholders from 46 countries in all six regions of WHO. Most DFIs present three essential features of WHO’s definition for DFI, that is, centering on the needs of persons living with dementia (PWD), multi-sector collaboration, and physical and social environmental changes. Over 70% participants reported their DFIs targeted PWD and included PWD as important partners. High-income countries tend to focus on enhancing professional capacity and environmental adaptation, while low-middle income countries prioritize dementia awareness campaigns. This corresponds to the reported disparities in levels of inclusion of PWD in societies, support to PWD, and service access for PWD found between low-middle income countries and high-income countries.

